# Association of Cannabis Use and Risks of Oral Cancer: A Systematic Review and Meta‐Analysis

**DOI:** 10.1002/hsr2.73499

**Published:** 2026-11-10

**Authors:** Asif Jan, Shubham Kumar, Bushra Waheed, Pratima Pandey, Huma Sultana, Daniel Dubwa

**Affiliations:** ^1^ Department of Pharmacy University of Peshawar KP Pakistan; ^2^ Saidu Group of Teaching Hospitals, Saidu Sharif Swat KP Pakistan; ^3^ District Headquarter Hospital Charsadda Charsadda KP Pakistan; ^4^ Dental Research Cell, Dr. D. Y. Patil Dental College & Hospital Dr. D. Y. Patil Vidyapeeth (Deemed To Be University) Pune India; ^5^ Department of Pharmacy University of Swabi Swabi KP Pakistan; ^6^ Department of Pharmacy Practice National Institute of Pharmaceutical Education and Research S.A.S Nagar India; ^7^ Department of Pharmaceutical Chemistry, Faculty of Pharmacy MS Ramaiah University of Applied Sciences Bangalore India; ^8^ Department of Pharmacy Makerere University Kampala Uganda

**Keywords:** association, cannabis use, meta‐analysis, oral cancer, public health, PubMed

## Abstract

**Background:**

Oral cancer is a major health concern globally, with its etiology linked to factors such as tobacco use, alcohol consumption, and HPV infection. With changing lifestyle behaviors and the increased legalization of cannabis, its potential role in oral carcinogenesis has garnered attention. This systematic review and meta‐analysis aims to evaluate the association between cannabis use and the risk of developing oral cancer.

**Methods:**

A systematic search was conducted across PubMed, EMBASE, and Web of Science to identify studies up to August 15, 2025. Inclusion criteria encompassed adult participants and observational studies on cannabis use and oral cancer risk. Meta‐analysis utilized random‐effects models to compute pooled odds ratios (ORs) in R software version 4.4. Newcastle Ottawa scale was used for quality assessment.

**Results:**

From 1,063 records, 11 studies met the inclusion criteria with a total sample size of 245,594 participants. The meta‐analysis revealed a pooled OR of 0.481 (95% CI: 0.306 to 0.757), suggesting a potential reduced risk of oral cancer with cannabis use, particularly noted in case‐control studies (pooled OR: 0.455, 95% CI: 0.291 to 0.713). However, cohort study showed no significant association (pooled OR: 2.51, 95% CI: 0.244 to 25.823), and high heterogeneity was observed across studies.

**Conclusion:**

Current evidence does not support cannabis use as an independent risk factor for oral cancer. Although some case‐control studies suggested an inverse association, this finding was not supported by cohort studies and should be interpreted cautiously given the substantial heterogeneity and potential for residual confounding. Further prospective studies are needed to clarify this relationship.

## 1 Introduction

Oral cancer, encompassing malignancies of the lips, tongue, floor of the mouth, and oropharynx, is a significant global health concern [1, 2]. As the sixth most common cancer worldwide, oral cancer accounts for nearly 377 713 new cases annually, with a high mortality rate associated with late‐stage diagnosis and aggressive tumor behavior [3]. The primary etiological factors for oral cancer include tobacco use, alcohol consumption, and human papillomavirus (HPV) infection, all of which have been extensively studied. However, as lifestyle behaviors evolve, researchers have begun to investigate the potential role of other substances, such as cannabis, in contributing to the development of oral cancer.

Cannabis, derived from the *Cannabis sativa* plant, is one of the most widely used psychoactive substances globally, particularly for recreational and medicinal purposes [4]. Despite longstanding prohibitions in many countries, cannabis use has been decriminalized or legalized in a growing number of regions, reflecting shifting public attitudes and increasing recognition of its therapeutic potential [5, 6]. As of 2023, approximately 188 million people worldwide are estimated to use cannabis, with significant increases in regions where legal frameworks permit its use [7]. While cannabis has demonstrated efficacy in the treatment of conditions such as chronic pain, chemotherapy‐induced nausea, and epilepsy, concerns remain about its potential adverse health effects, particularly with long‐term, heavy use [8].

One of the major concerns associated with cannabis use is its potential carcinogenicity [9]. Like tobacco smoke, cannabis smoke contains numerous harmful chemicals, including polycyclic aromatic hydrocarbons (PAHs), tar, and volatile organic compounds [10, 11]. Many of these substances are known to be carcinogenic and have been implicated in the development of various cancers, particularly of the respiratory tract. Despite these parallels between tobacco and cannabis, the relationship between cannabis use and the development of cancers, particularly oral cancers, remains underexplored and inconclusive.

Several epidemiological studies have sought to investigate the association between cannabis use and oral cancer risk, yet the findings remain inconsistent. Some studies suggest a potential link between chronic cannabis use and increased risk of developing oral cancer, positing that the presence of carcinogens in cannabis smoke could lead to DNA damage and cellular mutations [12]. However, other studies report no significant association, with some even suggesting that cannabis use may not pose a considerable risk for oral carcinogenesis [13, 14]. This divergence in findings may be attributed to differences in study design, sample size, cannabis consumption patterns, and confounding factors such as concurrent tobacco or alcohol use, making it difficult to draw definitive conclusions. Furthermore, cannabis use is often accompanied by other lifestyle behaviors, such as smoking tobacco and drinking alcohol, both of which are well‐established risk factors for oral cancer. This co‐use complicates the ability to isolate the specific impact of cannabis on oral cancer risk. Additionally, the method of cannabis consumptionwhether smoked, vaporized, or ingested may influence its potential carcinogenic effects. Smoking cannabis, in particular, exposes the oral cavity to high temperatures and direct exposure to combustion products, which may exacerbate mucosal damage and contribute to carcinogenesis.

To address the current gaps in knowledge, this systematic review and meta‐analysis aimed to comprehensively assess the association between cannabis use and the risk of developing oral cancer. By synthesizing data from observational studies, we seek to clarify whether cannabis use independently contributes to oral cancer risk, beyond the effects of other known risk factors such as tobacco and alcohol.

## 2 Methods

This systematic review and meta‐analysis follows the guidelines outlined by the Preferred Reporting Items for Systematic Reviews and Meta‐Analyses (PRISMA) (Supporting Information: Table S1) [15]. The objective of this review is to investigate the association between cannabis use and the risk of developing oral cancer. No prospective protocol registration was performed for this systematic review and meta‐analysis.

### 2.1 Search Strategy

A systematic search of electronic databases, including PubMed, EMBASE, and Web of Science, was conducted to identify peer‐reviewed articles published up to August 15 2025. The search included both observational and case‐control studies that examined the relationship between cannabis use and oral cancer. The search terms were developed using keywords and Medical Subject Headings (MeSH) related to cannabis use, oral cancer, and epidemiological study designs. The search strategy included combinations of the following terms: “cannabis,” “marijuana,” “oral cancer,” “oral malignancy,” “head and neck cancer,” “case‐control,” and “cohort studies.” Boolean operators (AND, OR) were used to refine the search. No restriction was kept on type of article or language in the search strategy. The detailed search strategy has been displayed in Supporting Information: Table S2.

### 2.2 Eligibility Criteria

The inclusion criteria for this systematic review and meta‐analysis were as follows: studies were eligible if they were case‐control, cohort, or cross‐sectional studies investigating the association between cannabis use and the risk of developing oral cancer. Eligible studies had to include adult participants aged 18 years or older who reported cannabis use, regardless of the frequency or method of consumption. The primary outcome of interest was the development of oral cancer, including cancers of the lips, tongue, floor of the mouth, and oropharynx. Cannabis use, whether smoked, vaporized, or ingested, had to be reported as a primary or secondary exposure variable. Only articles published in English were considered.

Exclusion criteria were established to maintain focus on the specific relationship between cannabis use and oral cancer. Studies focusing on other head and neck cancers that were not classified as oral cancer were excluded. Additionally, reviews, meta‐analyses, case reports, and editorials were not included in the analysis. Studies that did not clearly differentiate between cannabis use and co‐occurring risk factors such as tobacco and alcohol were excluded to avoid confounding effects. Finally, non‐human studies were also excluded from this review.

### 2.3 Study Selection

Two independent reviewers conducted the screening process in two stages: first by title and abstract, followed by full‐text screening. Any discrepancies in study selection were resolved through discussion or consultation with a third reviewer. Inter‐reviewer agreement was assessed using Cohen's kappa coefficient at both the title/abstract screening stage and the full‐text eligibility assessment stage.

### 2.4 Data Extraction and Quality Assessment

Data extraction was conducted independently by two reviewers using a standardized data extraction form to ensure consistency and minimize bias. The information extracted from each study included the following: study characteristics such as the author(s), year of publication, country where the study was conducted, study design, sample size, race, ethnicity, and the number of male and female participants. Additionally, participant characteristics were recorded, including age, gender, history of cannabis use, tobacco and alcohol consumption, and other potential confounding variables that could influence the outcomes. Risk estimates, including odds ratios (OR) and their corresponding 95% confidence intervals (CIs), were also extracted, taking into account both adjusted and unadjusted estimates reported in the studies.

The quality of each study was assessed independently by two reviewers using the Newcastle‐Ottawa Scale (NOS) for observational studies. The scale evaluates studies based on three domains: selection of study groups, comparability of groups, and ascertainment of exposure and outcomes. Studies were classified as having low, moderate, or high quality based on their scores. Any disagreements in quality assessment were resolved by consensus.

### 2.5 Statistical Analysis

The meta‐analysis was conducted using R software version 4.4. Pooled risk estimates for the association between cannabis use and oral cancer were calculated using random‐effects models to account for heterogeneity between studies. Pooled ORs and 95% CIs were calculated. Adjusted ORs were used in the meta‐analysis. Heterogeneity was assessed using the Cochrane *Q* test and quantified using the I^2^ statistic, with values greater than 50% indicating substantial heterogeneity. We calculated and reported the 95% prediction interval alongside the pooled effect estimate [16]. Prediction interval provides an estimate of the range within which the true effect of a future comparable study may lie and offers a more informative assessment of the uncertainty associated with the observed between‐study heterogeneity.

Mendelian Randomization studies were not included in the quantitative meta‐analysis because their effect estimates are derived from genetic instrumental variable analyses and are not directly comparable to observational effect estimates obtained from case‐control or cohort studies. Such studies were instead synthesized narratively as complementary evidence.

### 2.6 Certainity of Evidence Assessment

The certainty of evidence for the primary outcome was assessed using the Grading of Recommendations Assessment, Development and Evaluation (GRADE) framework. Evidence from observational studies was initially rated as low certainty and subsequently downgraded based on risk of bias, inconsistency, indirectness, imprecision, and publication bias.

## 3 Result

### 3.1 Literature Search

A total of 1,063 records were identified through database searches from PubMed (*n* = 293), Embase (*n* = 508), and Web of Science (*n* = 262). After removing 315 duplicate records, 748 records remained for screening. Of these, 650 records were excluded based on title and abstract screening. A total of 98 reports were sought for full‐text retrieval, and all were successfully retrieved. After assessing the 98 full‐text articles for eligibility, 87 were excluded for the following reasons: 55 studies did not report the outcome of interest (oral cancer), and 27 studies did not focus on cannabis as the exposure of interest. As a result, 11 studies [12, 13, 14, 17, 18, 19, 20, 21, 22, 23, 24] were included in the qualitative synthesis, with 10 studies being eligible for the quantitative meta‐analysis (Figure 1). Inter‐reviewer agreement was high throughout the study selection process. The Cohen's kappa coefficient for title and abstract screening was κ = 0.87, indicating almost perfect agreement between reviewers. For full‐text eligibility assessment, the Cohen's kappa coefficient was κ = 0.91, also demonstrating almost perfect agreement.

**Figure 1:**
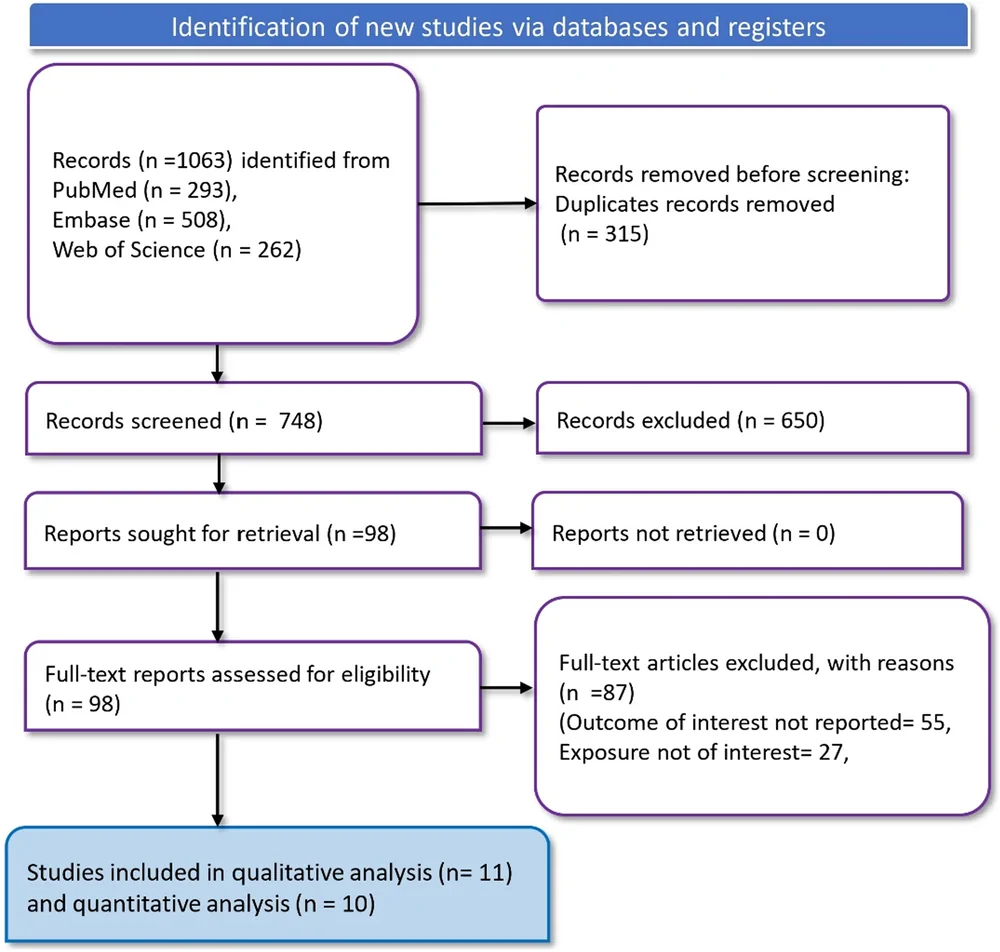
PRISMA flowchart depicting article selection and screening process.

### 3.2 Characteristics of Included Studies

The characteristics of included studies are given in Table 1. A total of 11 studies were included, covering a range of geographical regions and study designs. Among the included studies, four studies were conducted in the United States, two in the United Kingdom, one in Morocco, and four studies were international collaborations involving multiple countries. Six of the studies followed a case‐control design, while two were cohort studies, two were observational studies, and one was a cross‐sectional study. The total sample size across all studies was 245,594 participants, with individual study sizes ranging from 160 to 231,730 participants. The studies varied in their population characteristics, with most providing data on age, gender distribution, and race/ethnicity. For instance, Llewellyn et al. (2004) reported a predominantly White UK population, while Hashibe et al. (2006) included a more diverse sample of Non‐Hispanic Whites, African‐Americans, and Hispanics in the U.S. The studies also provided detailed information on the proportion of males and females, with the percentage of male participants ranging from 52% to 77% across the studies. The studies examined various confounders, including tobacco use, alcohol consumption, age, gender, race, socioeconomic status, and lifestyle factors such as dietary habits. Most studies adjusted their analyses for these confounders, aiming to isolate the effect of cannabis use on oral cancer risk. Key findings across studies were somewhat mixed, with some reporting a weak or no association between cannabis use and oral cancer, while others, such as Gallagher et al. (2024), suggested a higher risk of oral cancer associated with cannabis use. Sensitivity to tobacco and alcohol use was a common focus, given their well‐established role in oral carcinogenesis. The quality assessment of the studies is given in Supporting Information: Table S3.

**Table 1:** Characteristics of included studies.

Study	Country	Study design	Total patients	Case (n)	Control (n)	Age, mean (SD)	Male: n (%)	Female: n (%)	Race: n (%)	Adjusted cofounders	Key finding
Llewellyn 2004 [13]	UK	Case‐control study	323	116	207	Male = 39.3 (5.6) Female = 38.2 (5.9)	Case = 65 (56) Control = 119 (60)	Case = 51 (44) Control = 78 (40)	Whites UK: Case = 105 (91) Control = 172 (84) Blacks: Case = 2 (2) Control = 12 (5) Asian/Indian Case = 3 (2) Control = 5 (3) Others: Case = 6 (5) Control = 18 (9)	Tobacco habits, alcohol consumption, social class, ethnicity	Cannabis is not associated with Oral cancer
Hashibe 2006 [14]	USA	Case‐Control Study	1343	303	1040	NA	Case = 233 (77) Control = 623 (60)	Case = 70 (23) Control = 417 (40)	Non‐Hispanic Whites case = 192 (63) without Ca = 192 (63) African‐Americans: Case = 32 (11) Control = 102 (10) Hispanics Case = 51 (17) Control = 204 (20) Others Case = 28 (9) Control = 99 (10)	Cigarette smoking, Alcohol consumption, Age and gender, Socioeconomic status, Ethnicity, Other lifestyle factors	Association between cannabis use and oral cancers, even with heavy or long‐term use, is weak and may be undetectable
Llewellyn 2004 [17]	UK	Case‐Control Study	144	53	91	Case = 38.5 (7.0) Control = 39.2 (6.7)	Case = 28 (53) Control = 45 (49.4)	Case = 25 (47.1) Control = 46 (50.5)	Whites UK Case = 40 (76) Control = 71(78) White others Case = 7 (13) Control = 4 (4) Asians Case = 2 (4) Control = 5 (5) Blacks Case = 2 (4) Control = 8 (9) Others Case = 0 Control = 2 (2)	Cigarette smoking, Alcohol consumption, Age, Gender, Ethnicity	Cannabis is not associated with Oral cancer
Niu 2024 [18]	European	Observational study (Mendelian Randomization)	4151	1223	2928	NA	NA	NA	NA	Cigarette smoking, Alcohol consumption, Age, Gender, Ethnicity, Socioeconomic status, Dietary factors	Cannabis is not associated with Oral cancer
Rosenblatt 2004 [19]	USA	Case‐Control Study	1022	407	615	NA	Case = 70.8 Control = 71.5	Case = 29.2 Control = 28.5	Whites Case = 93.9 Control = v Blacks Case = 3.7 Control = 2.8 Others Case = 2.5 Control = 3.1	Sex, Education, Birth year, Alcohol consumption Cigarette smoking	Cannabis is not associated with Oral cancer in this large population‐based study
Gallagher 2024 [12]	California	Multicentre retrospective cohort study	231730	115865	115865	Case = 46.4 (16.8) Control = 46.7 (16.8)	Case = 61 272 (52.9) Control = 61 446 (53.0)	Case = 51598 (44.5) Control = 51314 (44.3)	American Indian or Alaska Native Case = 590 (0.5) Control = 500 (0.4) Asians Case = 975 (0.8) Control = 839 (0.7) Black/African ‐ american Case = 34926 (30.1) Control = 35004 (30.2) Native Hawaiian or Alaska Case = 896 (0.8) Control = 888 (0.8) Whites Case = 69550 (60.0) Control = 70053 (60.5) Other races Case = 2042 (1.8) Control = 1843 (1.6)	Cigarette smoking, Alcohol consumption, Age, Gender, Ethnicity	Cannabis use has been associated with higher risk of oral cancer
Shewale 2021 [20]	US	Case‐Control Study	747	249	498	NA	Case = 182 Control = 364	Case = 67 Control = 134	Whites Case = 229 Control = 458 Blacks Case = 17 Control = 34 Multiple or other races Case = 3 Control = 6 Hispanics Case = 4 Control = 7	Tobacco use, Alcohol use	Marijuana use may decrease the odds of HPV– negative OSCC
Marks 2014 [21]	NA	Case‐Control Study	4677	356	4321	NA	Case = 233(65.5) Control = 3208 (74.2)	Case = 123(34.5) Control = 1113 (25.8)	White non‐hispanic Case = 276 (77.5) Control = 2420 (56) Black Case = 7 (1.9) Control = 119 (2.9) Hispanic Case = 10 (2.8) Control = 86 (1.9) Asian Case = 8 (2.3) Control = 26 (0.6) Other Case = 2 (0.6) Control = 27 (0.6) Latin American Case = 53 (14.9) Control = 1643 (38)	Gender Race, Education, Tobacco use, Cigar/pipes, Pack years of tobacco smoking, Alcohol‐year	Marijuana use was found to have no effect on the association with oral tongue cancer
Liang 2009 [22]	US	Case‐Control Study	160	160	NA	59.68 (11.41)	NA	NA	NA	Age, Gender, Race, Education, Family history of cancer, Alcohol consumption	Marijuana use was associated with an attenuated risk of oral cancer
Kerouad 2021 [23]	Morocco	Cross‐sectional study	201	53	148	57.0 (15.4)	31 (58.5)	22 (41.5)	NA	Tobacco, Alcohol consumption	Female cannabis smokers may face higher risk of oral cancer
Berthiller 2009 [24]	NA	Pooled case‐control study	5996	981	5015	NA	NA	NA	NA	Age, Gender, Race, Education, Cigar smoking duration, Pack years of tobacco smoking, Smoking pipe duration, Alcohol duration	Marijuana is not associated with Oral cancer

### 3.3 Association of Cannabis Use and Risk of Oral Cancer

The meta‐analysis included both case‐control and cohort studies to examine the association between cannabis use and the risk of developing oral cancer. A total of 7 studies were included in the quantitative analysis, and the overall pooled OR for oral cancer was 0.481 (95% CI: 0.306 to 757), suggesting that cannabis use is associated with a reduced risk of oral cancer. The heterogeneity was found to be high (I^2^ = 97.6%). The case‐control studies showed a pooled OR of 0.455 (95% CI: 0.291 to 0.713), indicating a statistically significant reduction in oral cancer risk among cannabis users in these studies. However, heterogeneity was high (I^2^ = 98%, *p* < 0.01), suggesting variability in the effect estimates across the included case‐control studies. In contrast, the cohort study OR of 2.5 (95% CI: 0.244 to 25.82), which was not statistically significant (Figure 2). The Prediction interval was found to be from 0.113 to 2.047 indicating considerable uncertainty regarding the magnitude and direction of the association across different populations. Therefore, the pooled estimate should be interpreted with caution.

**Figure 2:**
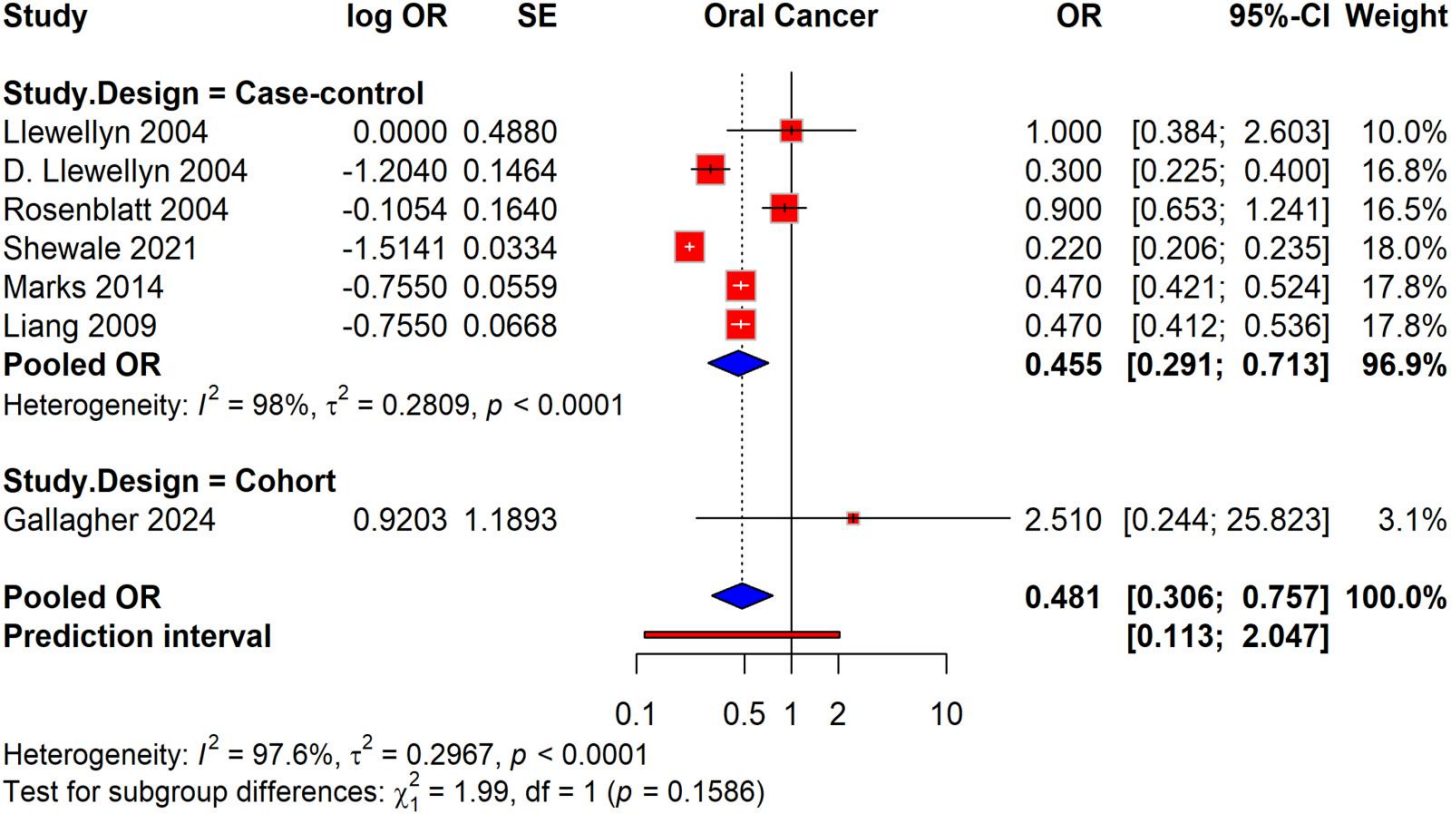
Forest plot depicting association of cannabis use and risk of oral cancer.

Following the exclusion of Niu et al. (2024) from the quantitative synthesis, the pooled OR changed from 0.653 in the original analysis to 0.481 (95% CI: 0.306–0.757), while heterogeneity increased from I^2^ = 88% to I^2^ = 97.6%. This change reflects the revised analytical dataset following the exclusion of the Mendelian randomization study.

### 3.4 Certainty of Evidence

The certainty of evidence for the association between cannabis use and oral cancer risk was assessed using the GRADE framework and was rated as very low. The certainty was downgraded because of very serious inconsistency across studies (I^2^ = 97.6%), serious indirectness related to variations in cannabis exposure assessment and outcome definitions, and serious imprecision due to limited cohort evidence and wide confidence intervals. Therefore, confidence in the pooled effect estimate is limited, and the true effect may be substantially different from the observed estimate (Supporting Information: Table S4).

## 4 Discussion

This systematic review and meta‐analysis aimed to explore the association between cannabis use and the risk of developing oral cancer. Although cannabis has become increasingly popular for both recreational and medicinal purposes, its potential long‐term adverse health effects, particularly its carcinogenic potential, remain a matter of concern. Oral cancer, a significant public health issue, is primarily associated with established risk factors such as tobacco use, alcohol consumption, and HPV infection. However, there is a growing need to investigate whether cannabis use independently contributes to oral carcinogenesis.

An important consideration when interpreting the present findings is the difference in evidence strength between study designs. Although the subgroup analysis of case‐control studies suggested an inverse association between cannabis use and oral cancer risk, this finding was not observed in cohort studies. Cohort studies are generally less susceptible to recall bias and temporal ambiguity and therefore provide a higher level of observational evidence. This distinction is particularly relevant for cannabis exposure, which is often self‐reported and may be influenced by underreporting, social desirability bias, or differential recall between cases and controls. Consequently, the inverse association observed in case‐control studies should be interpreted cautiously and may reflect residual confounding, selection bias, or exposure misclassification rather than a true protective effect. Taken together, the available evidence does not support cannabis use as an independent risk factor for oral cancer, although additional large prospective studies are needed to further clarify this relationship.

A notable limitation of this meta‐analysis was the substantial between‐study heterogeneity observed in the pooled analyses. The high I^2^ value indicates considerable variability in effect estimates across studies that is unlikely to be attributable to chance alone. Potential sources of heterogeneity include differences in study design, geographic location, participant characteristics, definitions and assessment of cannabis exposure, outcome classification, and the extent of adjustment for important confounders such as tobacco use and alcohol consumption. To further assess the impact of this variability, we calculated a 95% prediction interval, which demonstrated a wide range of plausible effects across future comparable populations. This finding suggests that the true association between cannabis use and oral cancer risk may differ substantially across settings and populations. Consequently, although the pooled estimate suggested a statistically significant association, the substantial heterogeneity limits confidence in the precision and generalizability of the summary effect. Therefore, the findings should be interpreted cautiously and should not be considered definitive evidence of either a protective or harmful effect of cannabis use on oral cancer risk.

According to the GRADE framework, the certainty of evidence for the association between cannabis use and oral cancer risk was rated as very low. This rating was primarily driven by substantial heterogeneity across studies, indirectness arising from heterogeneous exposure definitions and outcome classifications, and imprecision due to limited cohort evidence and wide confidence intervals. Consequently, confidence in the pooled estimate is limited, and the true effect of cannabis use on oral cancer risk may differ substantially from the observed findings.

Previous meta‐analyses have evaluated the relationship between cannabis use and various cancers. For instance, Gurney et al. [25] reported that current, long‐term, and frequent cannabis use are associated with an increased risk of developing testicular germ cell tumors (TGCT) compared to individuals who have never used cannabis. The strongest link was observed for non‐seminoma TGCT, with those using cannabis at least weekly having more than double the odds of developing non‐seminoma TGCT compared to non‐users (OR: 2.59, 95% CI 1.60–4.19). However, the evidence regarding the association between cannabis use and the risk of seminoma tumors was inconclusive in their analysis. Another meta‐analysis across 20 studies indicated a slightly significant correlation between smoking cannabis and the risk of lung cancer [26]. However, our results indicate that cannabis use was not associated with oral cancer.

The findings of this meta‐analysis have important clinical implications, particularly in light of the increasing legalization and decriminalization of cannabis worldwide. While the overall association between cannabis use and oral cancer risk appears to be weak, the high level of heterogeneity between studies suggests that the true relationship may vary depending on factors such as frequency of use, method of consumption, and the presence of other risk factors. Clinicians should continue to counsel patients on the potential risks associated with cannabis use, particularly in individuals who also smoke tobacco or consume alcohol, both of which are well‐established risk factors for oral cancer. In addition, given the increasing popularity of alternative methods of cannabis consumption such as vaporizing and edibles, it will be important to determine whether these methods carry the same risks as smoking. Vaporizing cannabis, for example, eliminates the combustion products that are present in smoke, but it is unclear whether vaporized cannabis poses the same risks for oral cancer. Similarly, the long‐term health effects of ingesting cannabis in edible form are not well understood and warrant further investigation.

The biological relationship between cannabis use and cancer development is complex and may involve both carcinogenic and potentially anti‐tumor mechanisms. Cannabinoid receptors, particularly CB1 and CB2, are expressed in a variety of tissues, including the oral mucosa and head and neck epithelium. Experimental studies have demonstrated that activation of these receptors may influence cellular proliferation, apoptosis, inflammation, angiogenesis, and tumor progression. In several in vitro and animal models, cannabinoids have been reported to exert anti‐proliferative and pro‐apoptotic effects on certain cancer cell lines, raising the possibility of anti‐tumor activity. However, these findings remain largely preclinical and their relevance to human oral carcinogenesis is uncertain. Moreover, cannabis smoke contains numerous combustion‐derived carcinogens, including polycyclic aromatic hydrocarbons and other toxic compounds that may promote DNA damage and malignant transformation. Therefore, the coexistence of potentially carcinogenic and anti‐tumor biological pathways may contribute to the inconsistent findings observed across epidemiological studies and underscores the need for further mechanistic and clinical research.

Residual confounding represents a major limitation of the current evidence base and may be one of the most plausible explanations for the inverse associations observed in some studies. Tobacco smoking and alcohol consumption are among the strongest established risk factors for oral cancer and frequently co‐occur with cannabis use, making it difficult to disentangle their independent effects. Although most included studies adjusted for tobacco and alcohol exposure, the extent and methodology of adjustment varied considerably, and residual confounding cannot be excluded. Furthermore, HPV infection is an important etiological factor for a subset of head and neck cancers, particularly oropharyngeal cancers, and was not consistently assessed or adjusted for across studies. Other factors, including socioeconomic status, oral hygiene practices, dietary habits, healthcare access, and lifestyle characteristics, may also have been inadequately measured or controlled. Given the observational nature of the available evidence, it remains difficult to determine whether the observed inverse associations reflect a true effect of cannabis use or differences in underlying risk profiles between users and non‐users. Consequently, residual confounding may partially or entirely account for the apparent reduction in oral cancer risk reported in some case‐control studies, further limiting confidence in causal interpretations of the findings and contributing to the overall uncertainty of the evidence.

Future research should prioritize large prospective cohort studies with long‐term follow‐up and standardized exposure assessment to better clarify the relationship between cannabis use and oral cancer risk. Detailed characterization of cannabis exposure, including product type, frequency, duration, cumulative exposure, and route of administration, will be essential for evaluating potential dose‐dependent and route‐specific effects. Future studies should also incorporate rigorous measurement and adjustment for major confounding factors, particularly tobacco use, alcohol consumption, HPV infection, and other relevant socioeconomic and lifestyle variables. In addition, further mechanistic research is needed to investigate the biological pathways linking cannabis use to oral carcinogenesis, including the potential carcinogenic effects of cannabis smoke and the role of cannabinoids in modulating tumor development. As cannabis legalization and use continue to expand globally, ongoing surveillance of oral cancer incidence in populations with varying patterns of cannabis exposure may provide valuable insights into its long‐term public health implications. Such methodological improvements will help reduce uncertainty, strengthen causal inference, and provide a more robust evidence base to inform clinical practice and public health policy. One of the key strengths of this meta‐analysis is its comprehensive approach to synthesizing the available evidence on cannabis use and oral cancer. By including studies from multiple geographical regions and study designs, this analysis provides a more complete picture of the relationship between cannabis use and oral cancer risk. Additionally, the use of random‐effects models to account for heterogeneity between studies ensures that the pooled estimates are more robust and reflective of the variability in the underlying studies.

However, several limitations should be considered when interpreting these findings. First, substantial heterogeneity was observed across the included studies, indicating considerable variability in effect estimates. Potential sources of heterogeneity include differences in study design, population characteristics, cannabis exposure assessment, outcome definitions, and adjustment for confounding variables. Although subgroup analyses by study design were performed, heterogeneity remained high, limiting confidence in the pooled estimates. In addition, the small number of cohort studies restricts the strength of conclusions that can be drawn from higher‐quality observational evidence. Second, residual confounding remains a major concern. Tobacco smoking and alcohol consumption are among the strongest established risk factors for oral cancer and frequently co‐occur with cannabis use, making it difficult to isolate the independent effect of cannabis. Although most studies adjusted for these factors, the extent and methodology of adjustment varied considerably. Furthermore, other important confounders, including HPV infection, socioeconomic status, oral hygiene practices, dietary factors, and healthcare access, were not consistently assessed. Consequently, residual confounding may partially or entirely explain the inverse associations reported in some studies. Third, the inability to evaluate specific routes of cannabis administration represents an important limitation. Combusted, vaporized, and orally consumed cannabis may differ substantially in their biological effects and carcinogenic potential; however, most studies either focused on smoked cannabis or did not report route‐specific exposure data in sufficient detail to permit subgroup analyses. As a result, cannabis use was evaluated as a broad exposure category, which may have contributed to the observed heterogeneity. Fourth, a formal dose‐response meta‐analysis could not be performed. Although some studies reported cumulative exposure measures such as joint‐years, the available data were inconsistently reported and lacked the detailed category‐specific information required for dose‐response modeling. Consequently, it was not possible to determine whether oral cancer risk varies according to the intensity or duration of cannabis exposure.

Another limitation relates to the potential overlap of participant populations among studies derived from the INHANCE consortium. Three included publications, Hashibe et al. (2006), Berthiller et al. (2009), and Marks et al. (2014), were based on INHANCE‐related datasets and may have included overlapping participants. Although these studies were treated as separate reports in the present synthesis, potential overlap cannot be completely excluded and may have resulted in disproportionate weighting of participants represented across multiple consortium publications. Consequently, the evidentiary contribution of these studies should be interpreted with caution, as correlated evidence from overlapping populations may influence the pooled estimate and the overall interpretation of the available literature. This potential dependency represents an additional source of uncertainty and should be considered when interpreting both the quantitative and narrative findings.

Finally, although major biomedical databases were searched, the review did not include CINAHL, the Cochrane Library, ClinicalTrials. gov, or the WHO ICTRP. Therefore, relevant unpublished, ongoing, or gray literature studies may not have been captured. Future research should prioritize large prospective cohort studies with standardized exposure assessment, detailed reporting of route and cumulative cannabis use, rigorous control of tobacco, alcohol, and HPV‐related confounding, and sufficient data to support dose‐response analyses.

## 5 Conclusion

Current evidence does not support cannabis use as an independent risk factor for oral cancer. Although some case‐control studies suggested an inverse association between cannabis use and oral cancer risk, this finding was not supported by cohort studies, which generally provide a higher level of observational evidence and are less susceptible to recall bias. Furthermore, the substantial between‐study heterogeneity and wide prediction interval indicate considerable uncertainty regarding the magnitude and direction of the association across different populations. Therefore, the observed inverse association should be interpreted cautiously, as it may reflect residual confounding, recall bias, selection bias, or exposure misclassification rather than a true protective effect. Additional well‐designed prospective cohort studies with standardized assessment of cannabis exposure are needed to clarify the relationship between cannabis use and oral cancer risk.

## Author Contributions

**Asif Jan:** investigation, conceptualization, writing – original draft, writing – review and editing, visualization, validation, methodology, funding acquisition, formal analysis, supervision. **Shubham Kumar:** conceptualization, investigation, validation, visualization, data curation, software. **Bushra Waheed:** investigation, conceptualization, methodology, validation, formal analysis. **Pratima Pandey:** investigation, conceptualization, methodology, validation, formal analysis. **Huma Sultana:** validation, conceptualization, writing – review and editing, formal analysis. **Daniel Dubwa:** visualization, validation, methodology, conceptualization, investigation, supervision, formal analysis, software.

## Funding

The authors have nothing to report.

## Conflicts of Interest

The authors declare no conflicts of interest.

## Transparency Statement

The lead Corresponding author(s) (Asif Jan and Daneil Dubwa) affirms that this manuscript is an honest, accurate, and transparent account of the study being reported; that no important aspects of the study have been omitted; and that any discrepancies from the study as planned have been explained.

## Supporting information


Supporting File

## Data Availability

The data are with the authors and available on request.
