# Supplementary material for: Association of Cannabis Use and Risks of Oral Cancer: A Systematic Review and Meta‐Analysis
Source: Health Sci Rep. 2026 Nov 10;9(11):e73499. doi: 10.1002/hsr2.73499 (PMC13752470; doi:10.1002/hsr2.73499)
Supplement: Supplementary file 1 [file HSR2-9-e73499-s001.docx]

**SUPPLEMENTARY MATERIALS**

**Table S1.** PRISMA Checklist

| **Section and Topic** | **Item #** | **Checklist item** | **Location where item is reported** |
| --- | --- | --- | --- |
| **TITLE** | | |  |
| Title | 1 | Identify the report as a systematic review. | 1 |
| **ABSTRACT** | | |  |
| Abstract | 2 | See the PRISMA 2020 for Abstracts checklist. (made as per the Journal guidelines) | 2 |
| **INTRODUCTION** | | |  |
| Rationale | 3 | Describe the rationale for the review in the context of existing knowledge. | 3 |
| Objectives | 4 | Provide an explicit statement of the objective(s) or question(s) the review addresses. | 3 |
| **METHODS** | | |  |
| Eligibility criteria | 5 | Specify the inclusion and exclusion criteria for the review and how studies were grouped for the syntheses. | 3 |
| Information sources | 6 | Specify all databases, registers, websites, organisations, reference lists and other sources searched or consulted to identify studies. Specify the date when each source was last searched or consulted. | 3 |
| Search strategy | 7 | Present the full search strategies for all databases, registers and websites, including any filters and limits used. | Table S2 |
| Selection process | 8 | Specify the methods used to decide whether a study met the inclusion criteria of the review, including how many reviewers screened each record and each report retrieved, whether they worked independently, and if applicable, details of automation tools used in the process. | 4 |
| Data collection process | 9 | Specify the methods used to collect data from reports, including how many reviewers collected data from each report, whether they worked independently, any processes for obtaining or confirming data from study investigators, and if applicable, details of automation tools used in the process. | 4 |
| Data items | 10a | List and define all outcomes for which data were sought. Specify whether all results that were compatible with each outcome domain in each study were sought (e.g., for all measures, time points, analyses), and if not, the methods used to decide which results to collect. | 4, Table 1 |
|  | 10b | List and define all other variables for which data were sought (e.g., participant and intervention characteristics, funding sources). Describe any assumptions made about any missing or unclear information. | 4 |
| Study risk of bias assessment | 11 | Specify the methods used to assess risk of bias in the included studies, including details of the tool(s) used, how many reviewers assessed each study and whether they worked independently, and if applicable, details of automation tools used in the process. | 4, |
| Effect measures | 12 | Specify for each outcome the effect measure(s) (e.g. risk ratio, mean difference) used in the synthesis or presentation of results. | 5 |
| Synthesis methods | 13a | Describe the processes used to decide which studies were eligible for each synthesis (e.g. tabulating the study intervention characteristics and comparing against the planned groups for each synthesis (item #5)). | 5, Table 1 |
|  | 13b | Describe any methods required to prepare the data for presentation or synthesis, such as handling of missing summary statistics, or data conversions. | NA |
|  | 13c | Describe any methods used to tabulate or visually display results of individual studies and syntheses. | 5 |
|  | 13d | Describe any methods used to synthesize results and provide a rationale for the choice(s). If meta-analysis was performed, describe the model(s), method(s) to identify the presence and extent of statistical heterogeneity, and software package(s) used. | 5 |
|  | 13e | Describe any methods used to explore possible causes of heterogeneity among study results (e.g. subgroup analysis, meta-regression). | 5 |
|  | 13f | Describe any sensitivity analyses conducted to assess robustness of the synthesized results. | 5 |
| Reporting bias assessment | 14 | Describe any methods used to assess risk of bias due to missing results in a synthesis (arising from reporting biases). | NA |
| Certainty assessment | 15 | Describe any methods used to assess certainty (or confidence) in the body of evidence for an outcome. | NA |
| **RESULTS** | | |  |
| Study selection | 16a | Describe the results of the search and selection process, from the number of records identified in the search to the number of studies included in the review, ideally using a flow diagram. | 5, |
|  | 16b | Cite studies that might appear to meet the inclusion criteria, but which were excluded, and explain why they were excluded. | 5, Table 1 |
| Study characteristics | 17 | Cite each included study and present its characteristics. | Table-1 |
| Risk of bias in studies | 18 | Present assessments of risk of bias for each included study. | Table S3 |
| Results of individual studies | 19 | For all outcomes, present, for each study: (a) summary statistics for each group (where appropriate) and (b) an effect estimate and its precision (e.g. confidence/credible interval), ideally using structured tables or plots. | Table 1, |
| Results of syntheses | 20a | For each synthesis, briefly summarise the characteristics and risk of bias among contributing studies. | 5 |
|  | 20b | Present results of all statistical syntheses conducted. If meta-analysis was done, present for each the summary estimate and its precision (e.g. confidence/credible interval) and measures of statistical heterogeneity. If comparing groups, describe the direction of the effect. | 5, 6 |
|  | 20c | Present results of all investigations of possible causes of heterogeneity among study results. | 5, 6 |
|  | 20d | Present results of all sensitivity analyses conducted to assess the robustness of the synthesized results. | 7 |
| Reporting biases | 21 | Present assessments of risk of bias due to missing results (arising from reporting biases) for each synthesis assessed. | Page 8 |
| Certainty of evidence | 22 | Present assessments of certainty (or confidence) in the body of evidence for each outcome assessed. | NA |
| **DISCUSSION** | | |  |
| Discussion | 23a | Provide a general interpretation of the results in the context of other evidence. | 8 |
|  | 23b | Discuss any limitations of the evidence included in the review. | 8 |
|  | 23c | Discuss any limitations of the review processes used. | 8 |
|  | 23d | Discuss implications of the results for practice, policy, and future research. | 9 |
| **OTHER INFORMATION** | | |  |
| Registration and protocol | 24a | Provide registration information for the review, including register name and registration number, or state that the review was not registered. | 4 |
|  | 24b | Indicate where the review protocol can be accessed, or state that a protocol was not prepared. | 4 |
|  | 24c | Describe and explain any amendments to information provided at registration or in the protocol. | NA |
| Support | 25 | Describe sources of financial or non-financial support for the review, and the role of the funders or sponsors in the review. | 10 |
| Competing interests | 26 | Declare any competing interests of review authors. | 10 |
| Availability of data, code and other materials | 27 | Report which of the following are publicly available and where they can be found: template data collection forms; data extracted from included studies; data used for all analyses; analytic code; any other materials used in the review. | 10 |

**Table S2.** The adjusted search terms as per searched electronic databases

| Database | Search Query | Results |
| --- | --- | --- |
|  | | |
| PubMed | ("mouth neoplasms"[MeSH Terms] OR "mouth neoplasm*"[All Fields] OR "oral squamous cell carcinoma"[All Fields] OR "oral cancer"[Title/Abstract] OR "oral leukoplakia"[All Fields] OR "Oral Neoplasm"[All Fields] OR "mouth cancer*"[Title/Abstract] OR "Oral cavity cancer"[Title/Abstract] OR "Oral carcinoma"[Title/Abstract] OR "oral malignancy*"[Title/Abstract] OR "oral neoplasm*"[Title/Abstract] OR ("Oral"[All Fields] AND "cancer*"[All Fields]) OR ("Oral"[All Fields] AND "carcinoma*"[All Fields])) AND ("cannabis"[MeSH Terms] OR "cannabis*"[Title/Abstract] OR "marijuana"[Title/Abstract] OR "marihuana"[Title/Abstract] OR "dronabinol"[MeSH Terms] OR "dronabinol*"[Title/Abstract] OR "dronabinol"[MeSH Terms] OR "dronabinol"[All Fields] OR "tetrahydrocannabinol"[All Fields] OR "tetrahydrocannabinolic"[All Fields] OR "tetrahydrocannabinols"[All Fields] OR "cannabinoids"[MeSH Terms] OR "cannabinoid*"[All Fields] OR "Cannabi"[Title/Abstract] OR "Hemp Plant"[Title/Abstract] OR "ganja*"[Title/Abstract] OR "bhang*"[Title/Abstract] OR "hemp*"[Title/Abstract] OR "Cannabis sativa"[Title/Abstract] OR "Cannabis indica"[Title/Abstract] OR "hashish*"[Title/Abstract] OR "Weed"[Title/Abstract] OR "Mary Jane"[Title/Abstract] OR "Dope"[Title/Abstract] OR "Kush"[Title/Abstract] OR "Skunk"[Title/Abstract] OR "Reefer"[Title/Abstract] OR "marijuana extract*"[Title/Abstract]) | **294** |
|  | | |
| Embase | ('mouth neoplasms'/exp OR 'mouth neoplasms' OR 'oral squamous cell carcinoma'/exp OR 'oral squamous cell carcinoma' OR 'oral cancer':ti,ab OR 'oral leukoplakia'/exp OR 'oral leukoplakia' OR 'mouth cancer':ti,ab OR 'oral cavity cancer':ti,ab OR 'oral carcinoma':ti,ab OR 'oral malignancy':ti,ab OR 'oral neoplasm':ti,ab OR ('oral':ti,ab AND 'cancer':ti,ab) OR ('oral':ti,ab AND 'carcinoma':ti,ab)) AND ('cannabis'/exp OR 'cannabis' OR 'cannabis':ti,ab OR 'marijuana':ti,ab OR 'marihuana':ti,ab OR 'dronabinol'/exp OR 'dronabinol' OR 'dronabinol':ti,ab OR 'tetrahydrocannabinol':ti,ab OR 'cannabinoid'/exp OR 'cannabinoid' OR 'cannabinoid':ti,ab OR 'hemp plant':ti,ab OR 'ganja':ti,ab OR 'bhang':ti,ab OR 'hemp':ti,ab OR 'cannabis sativa':ti,ab OR 'cannabis indica':ti,ab OR 'hashish':ti,ab OR 'weed':ti,ab OR 'mary jane':ti,ab OR 'dope':ti,ab OR 'kush':ti,ab OR 'skunk':ti,ab OR 'reefer':ti,ab OR 'marijuana extract':ti,ab) | 516 |
|  | | |
| Web of Science | TS=("mouth neoplasms" OR "mouth neoplasm*" OR "oral squamous cell carcinoma" OR "oral cancer" OR "oral leukoplakia" OR "oral neoplasm" OR "mouth cancer*" OR "oral cavity cancer" OR "oral carcinoma" OR "oral malignancy*" OR "oral neoplasm*" OR ("Oral" AND "Cancer*") OR ("oral" AND "carcinoma*")) AND TS=("cannabis" OR "cannabis*" OR "marijuana" OR "marihuana" OR "dronabinol" OR "tetrahydrocannabinol" OR "tetrahydrocannabinolic" OR "tetrahydrocannabinols" OR "cannabinoids" OR "cannabinoid*" OR "Cannabi" OR "Hemp Plant" OR "ganja*" OR "bhang*" OR "hemp*" OR "Cannabis sativa" OR "Cannabis indica" OR "hashish*" OR "Weed" OR "Mary Jane" OR "Dope" OR "Kush" OR "Skunk" OR "Reefer" OR "marijuana extract*") | 262 |

**Table S3.** Quality assessment using Newcastle-Ottawa Scale

| **STUDY** | **SELECTION (max 4 points)** | | | | **COMPARABILITY (max 2 points)** | **OUTCOME (max 3 points)** | | | **SCORE (out of 9)** |
| --- | --- | --- | --- | --- | --- | --- | --- | --- | --- |
|  | Representativeness | Selection | Ascertainment | Demonstration of the outcome of interest was not present at start of study | Comparability the basis of the design or analysis | Assessment of outcome | Was follow-up long enough for outcomes to occur? | Adequacy of the follow-up |  |
| Llewellyn 2004 (1) | 1 | 1 | 1 | 1 | 1 | 1 | 1 | 0 | 7 |
| Hashibe 2006 (2) | 1 | 1 | 1 | 1 | 2 | 1 | 1 | 0 | 8 |
| Llewellyn 2004 (3) | 1 | 1 | 1 | 1 | 1 | 1 | 1 | 0 | 7 |
| Niu 2024 (4) | 1 | 1 | 1 | 0 | 1 | 1 | 1 | 1 | 8 |
| Rosenblatt 2004 (5) | 1 | 1 | 1 | 1 | 2 | 1 | 1 | 1 | 8 |
| Gallagher 2024 (6) | 1 | 1 | 1 | 1 | 1 | 1 | 1 | 0 | 7 |
| Shewale 2021 (7) | 1 | 1 | 1 | 1 | 2 | 1 | 1 | 0 | 8 |
| Marks 2014 (8) | 1 | 1 | 1 | 1 | 1 | 1 | 1 | 0 | 7 |
| Liang 2009 (9) | 1 | 1 | 1 | 1 | 1 | 1 | 1 | 1 | 8 |
| Kerouad 2021 (10) | 1 | 1 | 1 | NA | 2 | 1 | NA | NA | 6 |
| Berthiller 2009 (11) | 1 | 1 | 1 | 1 | 2 | 1 | 1 | 1 | 9 |

**Table S4: GRADE assessment**

| **Outcome** | **No. of Studies** | **Study Design** | **Certainty of Evidence (GRADE)** | **Explanation** |
| --- | --- | --- | --- | --- |
| Oral cancer risk associated with cannabis use | 7 observational studies | Case-control and cohort studies | ⨁◯◯◯  Very Low | Downgraded for very serious inconsistency (I²=97.6%), serious indirectness (heterogeneous exposure definitions), and serious imprecision (wide confidence intervals and limited cohort evidence). |

**Bibliography**

1.	Llewellyn CD, Linklater K, Bell J, Johnson NW, Warnakulasuriya S. An analysis of risk factors for oral cancer in young people: a case-control study. Oral oncology. 2004;40(3):304-13.

2.	Hashibe M, Morgenstern H, Cui Y, Tashkin DP, Zhang Z-F, Cozen W, et al. Marijuana use and the risk of lung and upper aerodigestive tract cancers: results of a population-based case-control study. Cancer Epidemiology Biomarkers & Prevention. 2006;15(10):1829-34.

3.	Llewellyn C, Johnson N, Warnakulasuriya K. Risk factors for oral cancer in newly diagnosed patients aged 45 years and younger: a case–control study in Southern England. Journal of oral pathology & medicine. 2004;33(9):525-32.

4.	Niu D, Li C, Qu H, Zheng Y. Does cannabis elevate cancer risk? Evidence from Mendelian randomization. Wiener Klinische Wochenschrift. 2024;136(11):311-8.

5.	Rosenblatt KA, Daling JR, Chen C, Sherman KJ, Schwartz SM. Marijuana use and risk of oral squamous cell carcinoma. Cancer research. 2004;64(11):4049-54.

6.	Gallagher TJ, Chung RS, Lin ME, Kim I, Kokot NC. Cannabis Use and Head and Neck Cancer. JAMA Otolaryngology–Head & Neck Surgery. 2024.

7.	Shewale JB, Pickard RK, Xiao W, Jiang B, Gillison ML. Independent association of marijuana use and poor oral hygiene with HPV–negative but not HPV–positive head and neck squamous cell carcinomas. Cancer. 2021;127(12):2099-110.

8.	Marks MA, Chaturvedi AK, Kelsey K, Straif K, Berthiller J, Schwartz SM, et al. Association of marijuana smoking with oropharyngeal and oral tongue cancers: pooled analysis from the INHANCE consortium. Cancer Epidemiology, Biomarkers & Prevention. 2014;23(1):160-71.

9.	Liang C, McClean MD, Marsit C, Christensen B, Peters E, Nelson HH, et al. A population-based case-control study of marijuana use and head and neck squamous cell carcinoma. Cancer Prevention Research. 2009;2(8):759-68.

10.	Kerouad J, Errihani H, Ouasmani F, Benazzouz B, Chbicheb S, Mesfioui A. Tumor site related factors in patients with upper aerodigestive tract cancer in Morocco. Annals of Cancer Research and Therapy. 2021;29(2):161-8.

11.	Berthiller J, Lee Y-cA, Boffetta P, Wei Q, Sturgis EM, Greenland S, et al. Marijuana smoking and the risk of head and neck cancer: pooled analysis in the INHANCE consortium. Cancer Epidemiology Biomarkers & Prevention. 2009;18(5):1544-51.
